# A novel stem cell therapy for hepatitis B virus-related acute-on-chronic liver failure

**DOI:** 10.1590/1414-431X20209728

**Published:** 2020-10-07

**Authors:** Bing Zhu, Shaoli You, Yihui Rong, Qiang Yu, Sa Lv, Fangjiao Song, Hongling Liu, Huaming Wang, Jun Zhao, Dongze Li, Wanshu Liu, Shaojie Xin

**Affiliations:** 1 Medical School of Chinese PLA, Beijing, China; 2 Liver Failure Treatment and Research Center, Fifth Medical Center of Chinese PLA General Hospital, Beijing, China; 3 Department of Infection and Liver Diseases, Peking University International Hospital, Beijing, China; 4 Department of Interventional Therapy, Fifth Medical Center of Chinese PLA General Hospital, Beijing, China; 5 Liver Transplantation Center, Fifth Medical Center of Chinese PLA General Hospital, Beijing, China

**Keywords:** Stem cell transplantation, Hepatic artery transfusion, Acute-on-chronic liver failure, Hepatitis B virus, Randomized controlled trial

## Abstract

The aim of this study was to propose a stem cell therapy for hepatitis B virus (HBV)-related acute-on-chronic liver failure (ACLF) based on plasma exchange (PE) for peripheral blood stem cell (PBSC) collection and examine its safety and efficacy. Sixty patients (n=20 in each group) were randomized to PE (PE alone), granulocyte colony-stimulating factor (G-CSF) (PE after G-CSF treatment), and PBSC transplantation (PBSCT) (G-CSF, PE, PBSC collection and hepatic artery injection) groups. Patients were followed-up for 24 weeks. Liver function and adverse events were recorded. Survival analysis was performed. PBSCT improved blood ammonia levels at 1 week (P<0.05). The level of total bilirubin, international normalized ratio, and creatinine showed significant differences in the 4th week of treatment (P<0.05). The survival rates of the PE, G-CSF, and PBSCT groups were 50, 65, and 85% at 90 days (P=0.034). There was a significant difference in 90-day survival between the PE and PBSCT groups (P=0.021). The preliminary results suggested that PBSCT was safe, with a possibility of improved 90-day survival in patients with HBV-ACLF.

## Introduction

Hepatitis B virus-related acute-on-chronic liver failure (HBV-ACLF) is one of the most common types of liver failure in China and represents many challenges in clinical treatment (1). The 3-month mortality rate of liver failure is up to 57.8%, and there are no effective therapies except liver transplantation (2). Nucleoside analog with strong virus inhibition effect can improve the survival of HBV-ACLF patients, but severe inflammatory necrosis and deficient regeneration ability of hepatocytes make liver transplantation the only effective treatment for some patients with liver failure (3). The serious discrepancy between the pressing need for liver transplantation and the shortage of organ donors urgently needs to be solved by a new treatment strategy (4,5).

Growing evidence suggests that stem cell therapy could be a promising strategy for the treatment of liver failure by inhibiting liver inflammation, necrosis, and subsequent fibrosis (6). These stem cells, including umbilical cord blood mesenchymal stem cells (UCBMSCs), bone mesenchymal stem cells (BMSCs), and peripheral blood stem cell (PBSCs), all have their strengths and weaknesses (6). The number of BMSCs is small, and these cells have to be cultured *in vitro* (7). The application of UCBMSCs is also limited because of ethical issues (8). Moreover, the time required to culture UCBMSCs may cause treatment delay (9). PBSCs, the earliest discovered and most studied stem cells, are autologous cells and abundant, and the potential risks of culture are few. PBSCs have been widely used in cell replacement therapy, gene therapy, and tissue engineering (10). PBSCs can not only differentiate into various hematopoietic system components, but also into hepatocytes ([Bibr B11] [Bibr B12] 1113). Animal experiments indicate that human PBSCs could migrate to the liver of animals and express human liver cytological markers (14,15). Autologous CD34+ cells and concentrated mononuclear cells can improve liver function and stimulate hepatocyte regeneration. Spahr et al. found hepatocyte nodules formed by CD34+ cells in the treatment of alcoholic hepatitis (16). Gordon et al. (17) extracted CD34+ PBSCs from granulocyte colony-stimulating factor (G-CSF)-mobilized blood and transfused them back after expansion *in vitro*, which improved liver function and relieved symptoms. Yannaki et al. (18) reported two cases with alcoholic decompensated cirrhosis treated by boost infusions of autologous mobilized PBSCs and showed a lasting amelioration during the clinical course. These studies provide theoretical support for the clinical application of PBSCs transplantation (PBSCT).

Many clinical trials using various sources for stem cell transplantation for end-stage liver disease treatment have been reported and show some certain clinical effects (19-21). Because of the poor coagulation functions in patients with liver failure (22), it is difficult to achieve hepatic artery intubation for stem cell transplantation. Furthermore, the ethical issues associated with embryonic stem cells and the limitations regarding the extraction of BMSCs are seriously delaying the application of stem cell therapy for liver diseases. Therefore, our group has proposed a unique method that combines plasma exchange (PE) for PBSCs collection, which can simplify the collection of stem cells and be adjusted easily to different treatment timing. Hence, the aim of the study was to propose a novel stem cell therapy for HBV-ACLF based on plasma exchange (PE) for peripheral blood stem cell (PBSC) collection. A randomized controlled trial is presented to examine its safety and efficacy.

## Material and Methods

### Study design

This is a preliminary randomized, open-label, parallel controlled phase I clinical trial that was conducted at the Fifth Medical Center of Chinese PLA General Hospital from January 2015 to December 2017 (ChiCTR-TRC-12003518). The study protocol and procedures were in line with the Helsinki Declaration and were approved by the Ethics Committee of the Fifth Medical Center of Chinese PLA General Hospital. Formal written informed consent was obtained from each patient.

### Patients

Sixty patients (n=20/group) were randomly divided into three groups according to a random number table generated by a computer: PE (PE alone), granulocyte colony-stimulating factor (G-CSF) (PE after G-CSF treatment), and PBSC transplantation (PBSCT) (G-CSF, PE, PBSC collection and hepatic artery injection). There was no blinding.

The inclusion criteria were: 1) providing informed consent; 2) 18 to 65 years of age; 3) serum HBsAg positive for >6 months; 4) diagnosis of HBV-ACLF (23,24); and 5) peripheral blood leukocytes >3.0×10^9^/L and platelets >50×10^9^/L.

Patients meeting the following criteria were excluded: 1) another liver disease (e.g., acute fatty liver of pregnancy, drug-induced hepatitis, or liver cancer); 2) complicated with other viral infections (e.g., anti-HAV IgM, anti-HCV, or anti-CMV antibody positive); 3) uncontrolled infections, shock, or active gastrointestinal bleeding; 4) complicated with severe disease such as uncontrolled heart, lung, kidney, etc. disease; 5) history of allergy to blood products, heparin, or protamine; and 6) any other conditions considered inappropriate for treatment by physicians.

### Intervention

**Figure 1:**
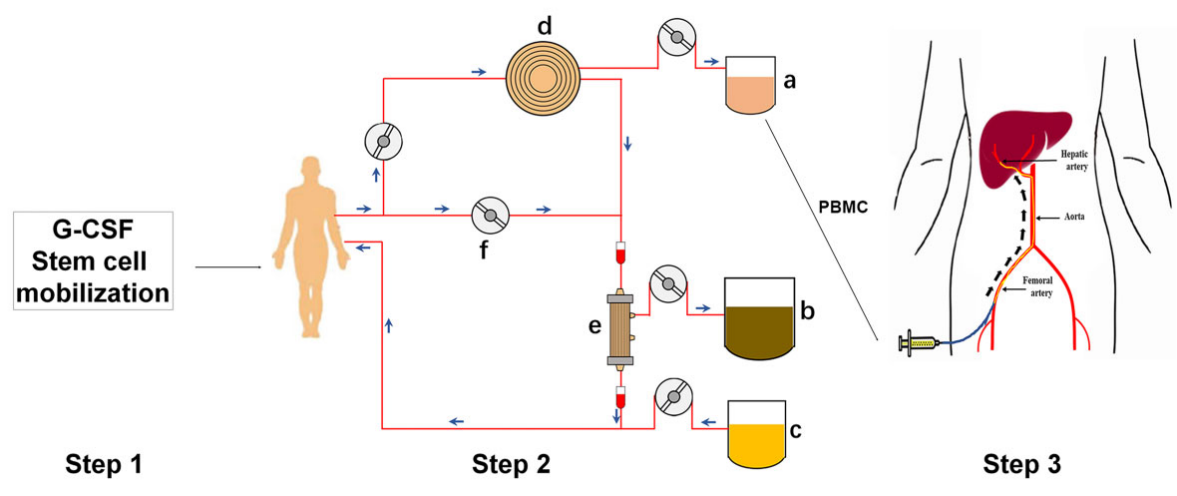
After mobilization using granulocyte colony-stimulating factor (G-CSF), the collection of peripheral blood mononuclear cells (PBMCs), including peripheral blood stem cells (PBSCs), was carried out using a self-made linkage device. The arrows point in the direction of blood flow. At the same time as plasma exchange, PBMCs were collected by the blood cell acquisition device. Then, the hepatic artery PBSC transplantation was performed. a) PBMCs; b) waste plasma obtained by replacement; c) fresh plasma; d) blood cell separator; e) plasma separator; f) pump.

All patients were given entecavir or tenofovir antiviral treatment after admission, regardless of whether they received antiviral treatment prior to admission. Patients in the PE group received basic treatment, including medical treatment and PE. Patients in the G-CSF group received basic treatment and G-CSF treatment. The patients were subcutaneously injected with G-CSF (5 μg/kg) for 4 days to mobilize bone marrow. The dose of G-CSF was decreased when the number of white blood cells (WBC) in peripheral blood was >30×10^9^/L. Patients in the PBSCT group received PE combined with stem cell collection after 4 days of G-CSF injection under basic medical treatment, using a methodological approach proposed by us for the first time. Harvesting of PBSCs was achieved through our self-developed collection linkage system, shown in Figure 1. The peripheral blood mononuclear cells (PBMCs) mobilized by recombinant (rh) G-CSF, including PBSCs, were collected during PE treatment. PBMCs were stored at 37°C and injected into the hepatic artery through interventional guidance and femoral artery puncture within 30 min. The total treatment time was about 3 h. The concentration of PBMCs was 10^9^-10^10^/L, and about 60-90 mL was injected.

### Follow-up and laboratory indexes

All patients were followed at baseline, 4 days, 1, 2, 4, 12, and 24 weeks. The main evaluation index was survival rate, as well as liver function, renal function, blood ammonia, Child-Turcotte-Pugh (CTP) score, and Model for End-Stage Liver Disease (MELD) score. Abdominal ultrasound was performed 3 days after transplantation to assess hepatic vessels. Any adverse event was recorded.

### CD34+ cell determination

The PBMCs collected by the system mentioned above were incubated with FITC-labeled CD34+ monoclonal antibody (BD Biosciences, USA) for 30 min at 4°C. The number of CD34+ cells was measured by flow cytometry (XL, Beckman Coulter, USA) after centrifugation (750 *g* for 30 min, 24^o^C) and washing. The exact methods have been published (25).

### Statistical analysis

All data were processed using SPSS 17.0 (IBM, USA). Continuous variables are reported as means±SD and were analyzed by ANOVA with the LSD test or the rank-sum test, as appropriate based on the Kolmogorov-Smirnov test. Categorical data are presented as frequencies and were analyzed using the chi-squared test. The survival rate was calculated by the Kaplan-Meier method and analyzed using the log-rank test. P<0.05 was considered statistically significant.

## Results

### Characteristics of the patients

**Figure 2:**
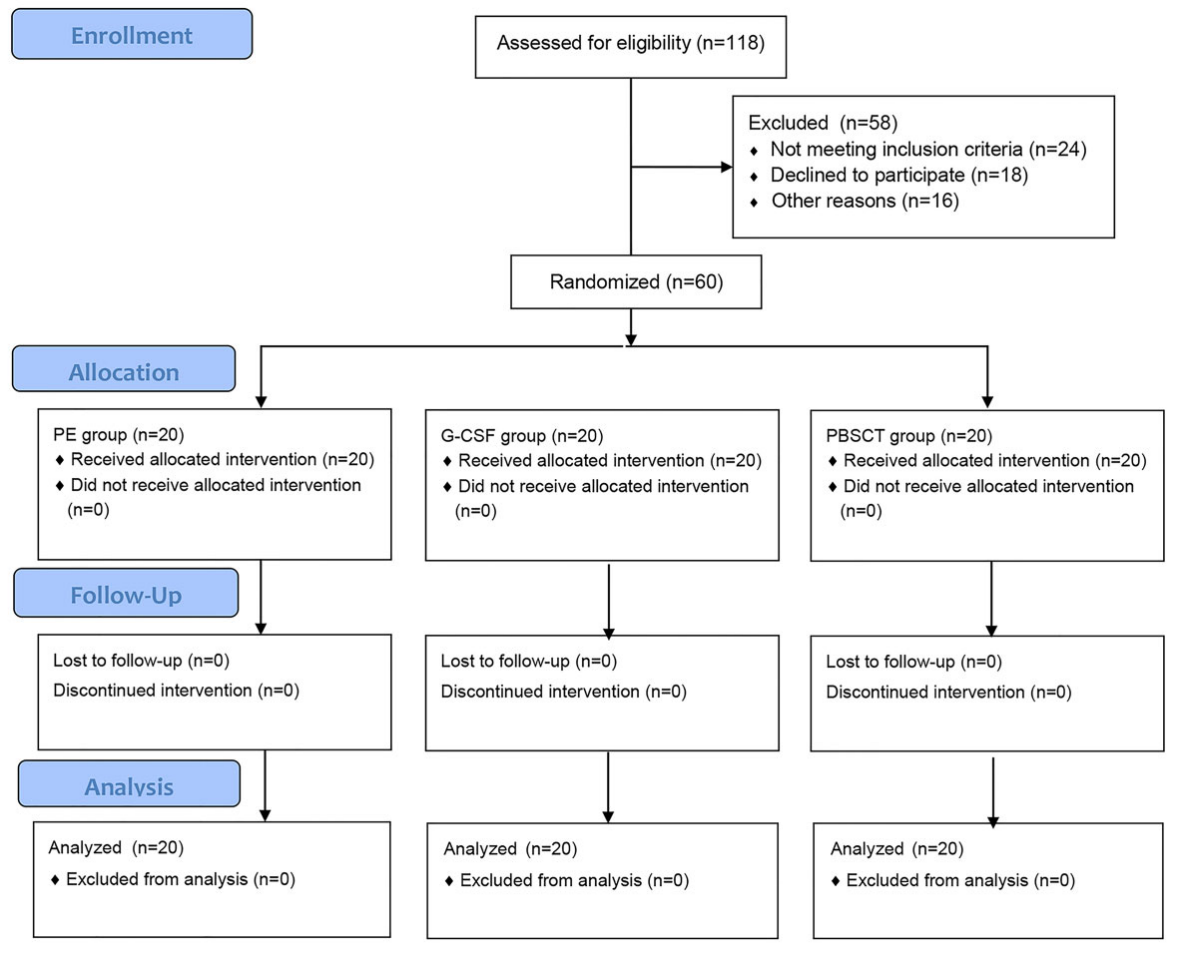
Study flow diagram. PE, plasma exchange. G-CSF, granulocyte colony-stimulating factor. PBSCT, peripheral blood stem cell transplantation.

Sixty patients (59 males and 1 female) were included in the study (Figure 2). There were no significant differences in baseline indicators, including age, biochemical indicators, international normalized ratio (INR), WBC, creatinine, α-fetoprotein, and HBV DNA among the three groups (Table 1). After six months of follow-up, the HBV DNA of the surviving patients gradually dropped below the detection threshold.

**Table 1:** Baseline characteristics of patients in the study population (n=20/group).

Characteristics	PE group	G-CSF group	PBCST group	P
Age (years)	43.95±9.32	45.10±7.14	40.10±8.15	0.143
Gender (males, %)	20 (100%)	19 (95%)	20 (100%)	1.000
WBC (10^9^/L)	6.62±2.58	6.03±2.54	5.95±2.55	0.666
ALT (U/L)	136.95±95.10	136.15±99.21	123.85±74.93	0.877
AST (U/L)	154.75±83.03	141.10±67.99	129.05±55.75	0.512
TBIL (μmol/L)	401.46±121.67	390.92±118.66	358.63±99.17	0.467
ALB (g/L)	29.70±4.96	31.35±3.72	30.70±2.99	0.423
CHE (U/L)	3557.20±1541.64	3348.15±1257.64	3925.80±968.07	0.475
AFP (ng/mL)	154.38±225.76	157.77±204.98	84.96±127.61	0.833*
INR	2.09±0.41	2.02±0.42	1.87±0.26	0.159
NH_3_ (μmol/L)	57.01±21.91	59.99±23.17	59.44±32.90	0.931
CRE (μmol/L)	99.50±33.79	107.20±68.18	89.65±18.95	0.734*
HBV DNA (log_10_)	4.83±1.64	4.45±1.60	4.60±1.52	0.709
CTP score	11.00±0.32	10.95±0.22	10.95±0.61	0.899*
MELD score	26.97±4.48	26.76±4.47	24.62±2.65	0.155

PE: plasma exchange; G-CSF: granulocyte colony-stimulating factor; PBCST: peripheral blood stem cell transplantation; WBC: white blood cells; ALT: alanine transaminase; AST: aspartate transaminase; TBIL: total bilirubin; ALB: albumin; CHE: cholinesterase; AFP: α-fetoprotein; INR: international normalized ratio; CRE: creatinine; HBV DNA: hepatitis B virus DNA; CTP score: Child-Turcotte-Plugh score; MELD: model for end-stage liver disease. Data are reported as means±SD. *Rank sum test was used for statistical analysis; one-way ANOVA was used for all others.

### Leukocyte monitoring

**Figure 3:**
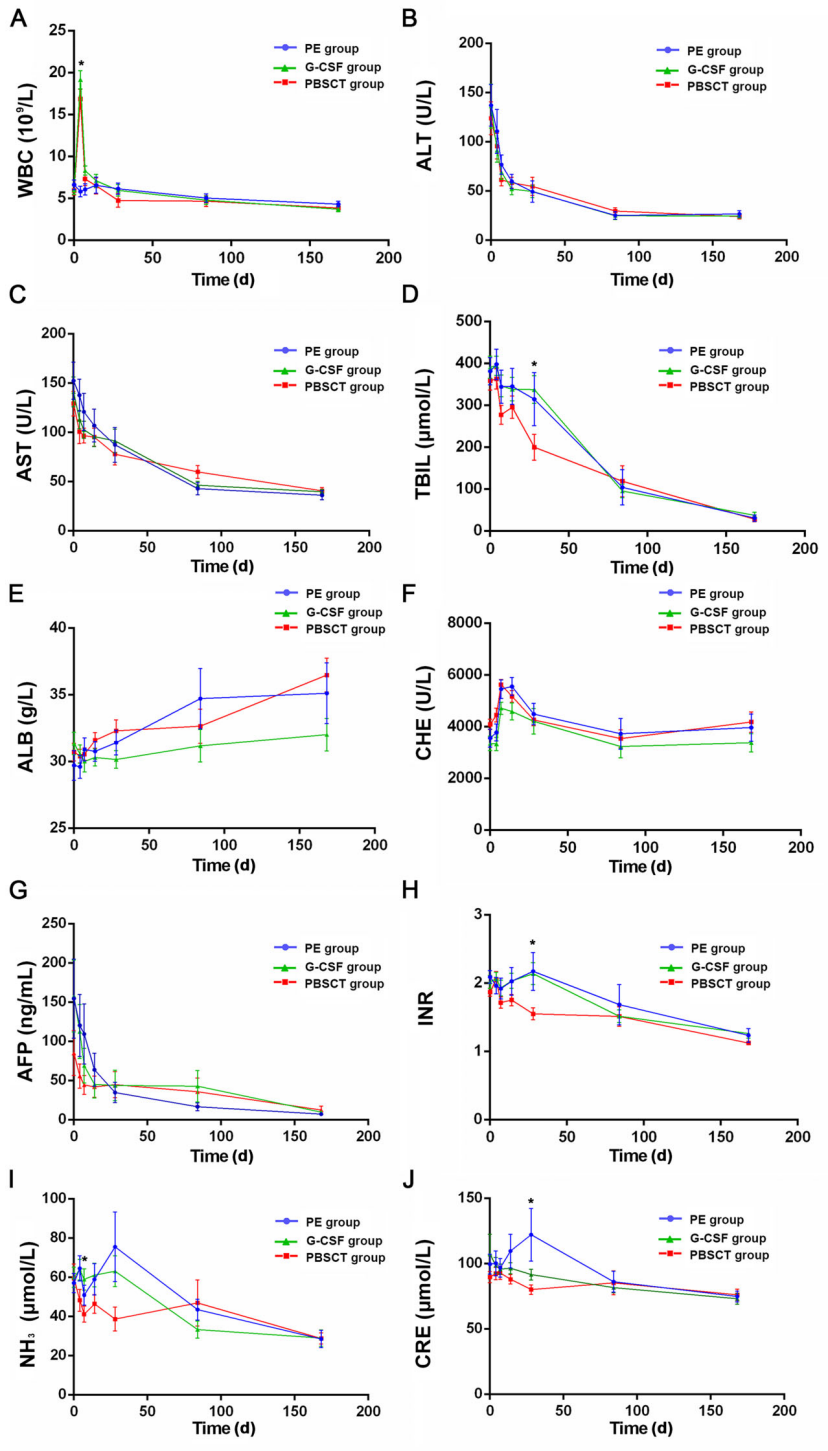
Changes of leucocyte levels and biochemical indexes in plasma exchange (PE), granulocyte colony-stimulating factor (G-CSF), and peripheral blood stem cell transplantation (PBSCT) groups. **A**, White blood cells (WBC) counts of G-CSF and PBSCT groups increased significantly on the fourth day of G-CSF treatment (*P<0.05 *vs* the PE group). Increased WBC levels returned to normal after G-CSF treatment (all P>0.05 from 1 week forward). **B**-**J**, The levels of ammonia (NH_3_) (**I**) showed significant differences in the first week (*P<0.05). The levels of alanine aminotransferase (ALT), aspartate aminotransferase (AST), total bilirubin (TBIL), albumin (ALB), cholinesterase (CHE), α-fetoprotein (AFP), international normalized ratio (INR), and creatinine (CRE) were also evaluated. Data are reported as means±SD. *P<0.05 (ANOVA with the LSD test).

The leukocyte levels are shown in Figure 3. After receiving G-CSF treatment, leukocyte levels in the G-CSF and PBSCT groups were increased significantly on the 4th day (both P<0.05 *vs* the PE group), then returned to normal at the subsequent monitoring points (P>0.05). In the PBSCT group, after collection, CD34+ cells were 5.50±1.82×10^8^/L.

### Biochemical indicators

Alanine transaminase, aspartate transaminase, total bilirubin, albumin, cholinesterase, α-fetoprotein, INR, NH_3_, and creatinine levels are reported in Figure 3. In the PBSCT group, PE was performed 4 days after G-CSF injection. After PE, INR (1.35±0.10) was significantly improved compared with baseline (1.87±0.26) (P<0.001). PBSCT improved blood ammonia levels at 1 week, but there were no significant differences at the subsequent time points. The levels of total bilirubin, INR, and creatinine showed significant differences in the 4th week of treatment (P<0.05). There was no significant increase in α-fetoprotein levels after cell transplantation. Albumin levels of the three groups showed a gradual improvement trend, but there was no statistically significant difference.

### Survival

**Figure 4:**
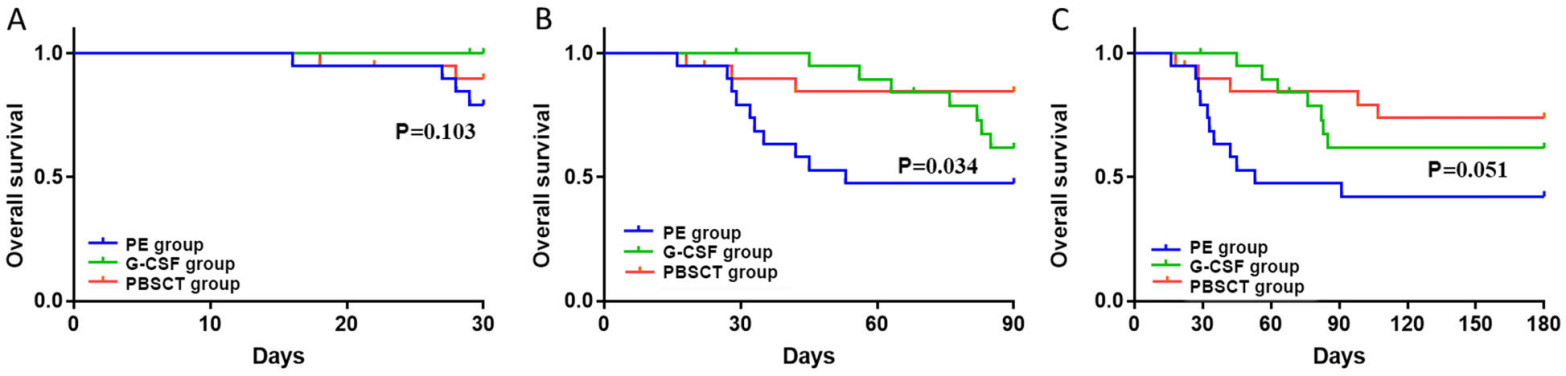
Survival curve analysis at 30, 90, and 180 days of plasma exchange (PE), granulocyte colony-stimulating factor (G-CSF), and peripheral blood stem cell transplantation (PBSCT) groups. **A**, There were no significant differences in 30-day survival among the three groups (P=0.103). **B**, There were significant differences in 90-day survival among the three groups (P=0.034). There was a significant difference in 90-day survival between the PE and PBSCT groups (P=0.021). **C**, There were no significant differences in 180-day survival among the three groups (P=0.051).

The survival rates of the PE, G-CSF, and PBSCT groups were 85, 100, and 90% at 30 days (P=0.103), 50, 65, and 85% at 90 days (P=0.034), and 45, 65, and 75% at 180 days (P=0.051) (Figure 4 and Table 2). There was a significant difference in 90-day survival between the PE and PBSCT groups (P=0.021). There was no difference in transplantation among the groups (Table 2).

**Table 2:** Deaths and transplantation in the three groups (n=20/group).

Characteristics	PE	G-CSF	PBCST
Death			
30 days	3	0	2
90 days	10	7	3
180 days	11	7	5
Transplantation			
30 days	1	1	1
90 days	1	2	1
180 days	1	2	1

PE: plasma exchange; G-CSF: granulocyte colony-stimulating factor; PBCST: peripheral blood stem cell transplantation.

### Safety

Only one patient (the second patient receiving treatment in the PBSCT group) had mild ecchymosis at the femoral artery puncture site, which was caused by inexperienced use of the femoral artery puncture compressor. After this event, nursing methods were emphasized and improved. Fever, ostealgia, fatigue, and other minor adverse effects often occurred during G-CSF injection, but they were tolerable and self-recovered. No serious adverse reaction was observed. No hepatic tumor, vascular obstruction, liver pain, or pulmonary embolism were found in the three groups during the 6-month follow-up.

## Discussion

Stem cell therapy can be used for recovery from end-stage liver disease. There are few reports of hepatic artery transfusion for stem cell therapy. Therefore, this study aimed to examine the safety and efficacy of stem cell therapy for HBV-ACLF based on PE for PBSC collection. This preliminary study suggested that PBSCT is safe, with a possibility of improved 90-day survival in patients with HBV-ACLF. Additional studies are needed to confirm the results.

In addition to the vital function of immune factors in the pathogenesis of HBV-ACLF, the effective regeneration of liver cells is an important factor affecting prognosis. Liver reserve capacity and hepatocyte regeneration is weakened due to the basic liver disease, especially cirrhosis, in patients with HBV-ACLF. The power of hepatocyte regeneration plays a major role in the outcome of HBV-ACLF; therefore, strengthening the hepatocytes regeneration ability to allow the effective replacement of the large hepatic necrosis is a key aspect of the treatment of HBV-ACLF in addition to antivirus drugs and immunoregulation (26,27). A study showed good outcomes at 24 months when using UCBMSCs in combination with entecavir (28).

Stem cells have great potential in the treatment of end-stage liver disease because of their self-renewal and directional differentiation abilities. Many studies have shown that stem cell therapy improves the survival of patients with end-stage liver disease by promoting hepatocyte regeneration and immunoregulation, but there are few reports about the treatment of liver failure (20,29-32). Many sources of stem cells have been proposed as sources for cell therapy (33). The use of UCBMSCs is impeded by medical ethics and unknown factors as allografts (6). BMSC application is also limited by multiple invasive bone marrow puncture and risk of contamination and cell mutation during the *in vitro* amplification, which could increase the cell count (7). On the other hand, hematopoietic stem cells have been widely used in cell replacement therapy, gene therapy, and tissue engineering (10).

In the present study, the innovative design of PE and stem cell collection linkage technology not only avoided multiple puncture and long-time treatment but also combined two extracorporeal circulations at the same time, which reduced the application of heparin and related adverse reactions. Stem cells were collected from the peripheral blood after mobilization by G-CSF. The results of the present study showed that the average number of CD34+ cells collected from each patient was 5.50±1.82×10^8^/L. Stem cell collection by this method is stable and easy.

No definite conclusion has been reached yet about the best transplantation route for stem cells. *In vivo* research showed that stem cell transplantation by portal vein infusion is more effective than via a peripheral vein, the hepatic artery, or an intrahepatic injection. For humans, portal vein infusion is difficult. Intravenous infusion is simple and easy, but tracer studies have found that transplanted cells mainly enter the lung at an early stage. Ten days later, most cells are in the spleen, and only 13.0-17.4% remain in the liver (34). Hepatic artery transplantation (HAT) has been suggested to be the best method for stem cell transplantation in the liver (35,36). HAT of stem cells has been reported in the treatment of end-stage liver disease but rarely in liver failure due to the greater risk of bleeding induced by coagulation disorders (32,37). In the present study, arterial puncture was conducted within 30 min after PE, which can significantly improve coagulation condition (INR improved from 1.87±0.26 pre-treatment to 1.35±0.10 post-treatment) and reduce bleeding risk. The safety of stem cell HAT was also suggested by the absence of local bleeding in the treatment group.

Hyperbilirubinemia, various inflammatory factors, and toxins in the plasma of patients with liver failure hamper the survival and growth of stem cells. Stem cell transplantation after PE is beneficial to the survival and proliferation of stem cells due to the improvement of the patient's internal environment. The main mechanisms of stem cell therapy include directional differentiation to repair the damaged liver, immunomodulatory, and inhibition of fibrosis (20,33,38). Total bilirubin, INR, and creatinine showed significant differences (P<0.05) in the PBSCT group at 4 weeks, suggesting that PBSCT might take some time to show observable clinical effects. The survival analysis indicated that there were no significant differences in the 30- and 180-day survival rates, but the difference between the 90-day survival curves of the PE and PBSCT groups was significant. The results suggested that patients in the PBSCT group may have benefited from the transplantation and showed a better survival rate as long as they survived the 30-day period. Nevertheless, this will have to be confirmed in future studies.

The lack of pathological support for hepatic directional differentiation and repair is a limitation of the present study. Nevertheless, our previous studies and the literature indicate that CD34+ cells can promote the growth of hepatocytes, differentiate into hepatocytes, and promote the recovery of liver inflammation in patients with chronic and acute liver failure by secreting hepatic growth factor, matrix metalloproteinase-9, stem cell factor, and interleukin-6 (25). In addition, the sample size was small, and the follow-up was short. Additional studies are needed to determine the exact clinical value of this novel approach involving PE and G-CSF for PBSCT.

The present study found that patients in the PBCST group had significantly better 90-day survival than the PE group (P=0.021). Although there was a difference in 90-day survival in the PBCST group compared to the GCS-F group (65 *vs* 85%), the difference was not statistically significant (P=0.198). This might be because of the small sample size, and it needs to be further confirmed by a larger sample size. Of course, in clinical work, the use of GCS-F alone is simple, easy to implement, and easily accepted by the patients. On the other hand, interventional transplantation, as an invasive operation, is more difficult to accept, which is the main reason why it was not easy to obtain informed consent for the expansion of this study. Nevertheless, it should be emphasized that the study was based on treatment with autologous peripheral blood stem cells and therefore compared with GCS-F treatment. With the development of stem cell technology, studies applying other stem cell therapies are becoming more and more common, and there is a need to explore viable methods for stem cell intrahepatic transplantation therapy. The present study provides an optimized strategy for stem cell therapy for intrahepatic transplantation in liver failure, demonstrating an innovative approach for subsequent studies.

Differences among groups may occur despite randomization. The baseline data showed that the individual indicators in the PBSCT group appeared to be better, but in the clinical setting and in previous studies, the small differences in specific values such as total bilirubin and cholinesterase are probably not clinically significant and should not be determinants of patient prognosis. Nevertheless, they were consistent with the diagnosis of HBV-ACLF, and the disease was considered clinically consistent among the three groups.

These preliminary results suggested that PBSCT was safe, with a possibility of improved 90-day survival in patients with HBV-ACLF. Additional studies are needed to confirm the results.
